# Behavior of Transplanted Multipotent Cells after in Vitro Transplantation into the Damaged Retina

**Published:** 2011

**Authors:** S.A. Sergeev, Y.V. Khramova, M.L Semenova, I.N. Saburina, N.V. Kosheleva

**Affiliations:** Biology Faculty, Lomonosov Moscow State University; Institute of General Pathology and Pathophysiology, Russian Academy of Medical Sciences

**Keywords:** stem cells, *in vitro*transplantation, retinal organotypic cultures, stem cell plasticity, retinal reparation

## Abstract

The use of stem cell technologies in retinal defect reparation therapy has produced beneficial results. Nowadays, numerous protocols exist which provide a neural differentiation of the stem cells transplanted into the retina. However, questions concerning the functional replacement of the missing retinal neurons by transplanted cells thus far remain unanswered. The organotypic culture protocol was used in this study in order to prove the possibility of transdifferentiation of bone marrow stromal cells (MMSCs) and neural stem/progenitor cells (NSPCs) from EGFP-positive mice and the functional integration of these cells. This technique enables a detailed characterization of cell behavior post-transplantation. Using atomic force microscopy, we reliably demonstrated the difference (*p *< 0.01) between the thickness of the outgrowths formed by glial and endothelial retina cells and the thickness of neurites and neuro-like transplanted MMSC outgrowths. MMSCs are also shown to form synapses up to 2.5 ± 0.06 µm in diameter on day 4 after the transplantation. Following electrical stimulation (20V, 0.5Hz, 200ms), clear depolarization of retinal neurons and their outgrowths is detected. It is shown that some of these GFP+ MMSCs, which changed their morphology after the transplantation in retinal explants to neuro-like MMSCs, are capable of depolarizing after exogenous stimulation.

## INTRODUCTION

Retinal changes caused by various exogenous damaging factors often lead to a partial or total loss of vision. In order to provide efficient therapy for retinal pathologies it is necessary to understand the processes involved in the emergence and progression of these pathologies, as well as the processes leading to retinal reparation. Methods for the transplantation of cells of wide differentiation potential – neural stem/progenitor cells (NSPCs) or bone marrow-derived mesenchymal stem cells (MMSCs) – are currently being developed in order to curb the progression of irreversible neurodegenerative processes in the retina. It is believed that, after they are introduced into a recipient’s organism, these cells not only actively migrate to the damaged site and replace the missing tissue elements with their differentiated descendants, but that they also secrete a broad range of trophic and regulatory factors that maintain the functionality of the damaged tissue and activate its own reparation systems [1].

Unfortunately, the theoretical and practical data thus far accumulated in the area of stem cell biology remains insufficient in order to reliably predict how efficient cell transplantation therapy can be. We are still some way from a complete understanding of the mechanisms that initiate reparation in a recipient’s tissue following the introduction of cells. Moreover, we still haven’t figured out means to control this process. In this context, it seems reasonable to design adequate models that are as close as possible to *in vivo* conditions, thereby enabling one not only to perform easy detection of cell migration, differentiation, and death at any stage following transplantation, but also to adjust the behavior of the transplanted cells. An organotypic explant culture of rat retina is one of such models. This type of culture is used to obtain specimens that are capable of retaining * in vitro * their initial cytoarchitectonics and cell structure for a long time [2]. 

Laser-induced local damage to retinal explants in the *in vitro* system is considered to be the most appropriate experimental model of retinal damage. This approach allows to inflict a strictly defined level of damage: i.e., to obtain reproducible results in an entire series of experiments. The use of this approach in experimental studies devoted to the simulation of neuronal network damage has a lot of potential, due to the fact that it allows to independently control the various parameters of laser radiation. 

## EXPERIMENTAL

**Obtainment of retinal cultures**

 The retinal explant culture was obtained from 4-day-old male Wistar rats. Eyeball dissection was performed according to the modified protocol [3]. After isolation of the retina, it was then placed on the surface of a 35 mm diameter Petri dish with the photoreceptor layer on top. 

Culturing was performed under standard conditions (+37˚С, 5% СО _2_ , 98% humidity) for 30 days in a DMEM/F12 medium (PanEco, С420/С600) containing glutamine (Sigma, G-8540) and insulin-transferrin-sodium selenite at 1 : 50 dilution (PanEco, F-065) supplemented with FCS ^TM^ 5% serum (HyClone, SH30109.03), the major fibroblast growth factor (10 ng/ml, Sigma, F0291), epidermal growth factor (10 ng/ml, Sigma, E9644), heparin, gentamicin at a concentration of 5 µl/ml (Sigma, G1264), additives N2 at 1 : 100 dilution (Gibco, 17502-048), and B27 at 1 : 50 dilution (Gibco, 17504044). 

**Damaging explant**

 A Zilos-tk ^TM^ infrared laser (Hamilton Thorne, 1480 nm, millisecond-long pulse duration, 300 mV intensity) heating the spot of laser focus to 150 ^0^ С was used as the damaging factor. A square with a 100 µm side length (within the average explant margin excrescence area) was selected as the object to be damaged. Damage was inflicted on the retina using 15 laser pulses (1000–3000 ms long) on day 14 of explant culturing. 

**Cell transplantation**

 Red bone marrow from EGFP-positive С57BL/6-Tg(ACTB-EGFP)/Osb/J mice (1 month) was used to obtain the MMSC culture. Red bone marrow specimens were taken from the shinbones and thigh bones of the animals immediately prior to euthanasia, according to the protocol [4]. The stroma of the bone marrow was removed by washing the bone cavity with an insulin syringe filled with the DMEM medium supplemented with antibiotic agents. The resulting suspension was centrifuged for 7 min at 1000  *g* . The supernatant liquid was poured away; the precipitate was then re-suspended and diluted with the culture medium to attain a density of 1 × 10 ^5^  cells/ml. The primary suspension cell culture from the stroma of the bone marrow was placed into culture dishes (60 mm in diameter); after 24 h, the cells were sub-cultured at a significantly low cell density (1–5 cells within the field of vision, 200x magnification). The cells were then cultured until colonies formed. 

**Fig. 1:**
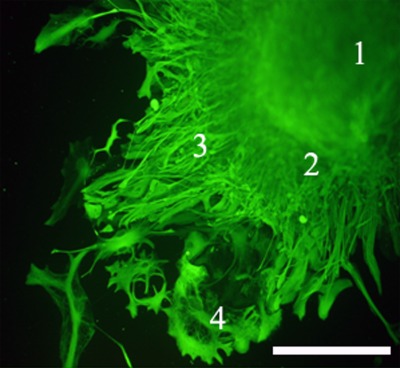
Migration of retinal explant cells ( *1* ) with the formation of multicellular close-in ( *2* ), middle ( *3* ), and monolayer distant ( *4* ) areas of explant margin excrescence. The scale bar is 300 µm.

The material taken from the subventricular zone of the brain of a 14-day-old С57BL/6-Tg(ACTB-EGFP)/Osb/J mouse embryo was used to culture NSPCs. After the brain was released from the meninges, it was placed into a DMEM medium supplemented with antibiotic agents and then subjected to mechanical deaggregation with a pipette until a homogeneous cell suspension was obtained. The cells were then centrifuged for 5 min at 1000  *g* . The supernatant liquid was poured away; the precipitate was subsequently re-suspended in the culture medium at a concentration of 1 × 10 ^6^ –2 × 10 ^6^  cells/cm ^2^ . The cells were cultured in the neuroepithelial stratum. 

The cells were injected with a glass microcapillary on a Nikon TE2000S inverted microscope equipped with hydraulic micromanipulators and Narishige injectors (MMO-202 ^ND^ , IM-9B, IM-H1, Narishige HD-21) into the zone of explant cell excrescence (0.1 µl of the culture medium) at a distance of 100, 1000, and 3000 µm from the damaged area. The efficiency of the protocols of cell transplantation into the retina imitating the suprachoroidal injection was assessed by placing the suspension of cells to be transplanted onto the external (photoreceptor) explant surface using a microinjector (medium volume being less than 5 µl). 

**Immunohistochemical analysis**

 The immunohistochemical analysis of the differentiation of the transplanted cells was performed by detecting neuronal and glial differentiation markers: βIII-tubulin – using anti-tubulin, beta III isoform antibody (Chemicon, MAB1637), and glial fibrillary acidic protein GFAP – using anti-glial fibrillary acidic protein antibody (Sigma, G9269), respectively. The expression of the neurite outgrowth markers GP-45 was also assessed using a G protein-regulated inducer of neurite outgrowth 2 antibody (Abcam, ab110898) and the capillary endothelial cell marker GSL-IB4 – isolectin from *Griffonia simplicifolia* IB4 (Sigma, L1509). 

**Cell imaging**

 The images of living cells were obtained using a Nikon TE2000S inverted microscope; the fluorescence images were taken using an Axsiovert 25 inverted fluorescence microscope. The spatial pattern of distribution of the transplanted cells in an explant was obtained via confocal laser scanning microscopy (Axsiovert 200LSM 510Meta Carl Zeiss microscope). 

**Statistical analysis**

 The reliability analysis of the results and statistical processing were performed using the STATISTICA 6.0 software. The existence and reliability of differences between the sample values of independent samplings was assessed using the nonparametric Kruskal–Wallis H test. The statistical significance of differences between the injected cell groups (MMSCs, NSPCs) was determined using the Student–Newman–Keuls test (ANOVA). 

**Atomic force microscopy (AFM)**

 AFM images were obtained on a Solver BIO Olympus atomic force microscope (NT-MDT, Russia) with a scanning range of 100 × 100 × 7 µm ^3^ , equipped with a system of capacity sensors and cantilever Veeco MSCT-AUHW (Veeco Instruments, USA) with a rigidity of 0.01–0.03 N/m. A laser operating at 650 nm was used to record the cantilever bending. The instrument was integrated into the inverted optical microscope in order for the cantilever to be close enough to the sample and establish the scanning sites. The images were processed using the Nova software (NT-MDT, Russia). 

## RESULTS AND DISCUSSION

Organotypic retinal explant cultures capable of surviving long-term *in vitro* were obtained during the first stage of our study. Tissue architectonics was retained during the entire culturing period (up to 30 days); the major cell types typical of an intact retina *in vivo* were present within the tissue [5] ( *Fig. 1* ). Thus, this explant culture is an adequate model of the developing neuroretina, enabling the retention of the micro-surrounding neural cells within. 

**Fig. 2:**
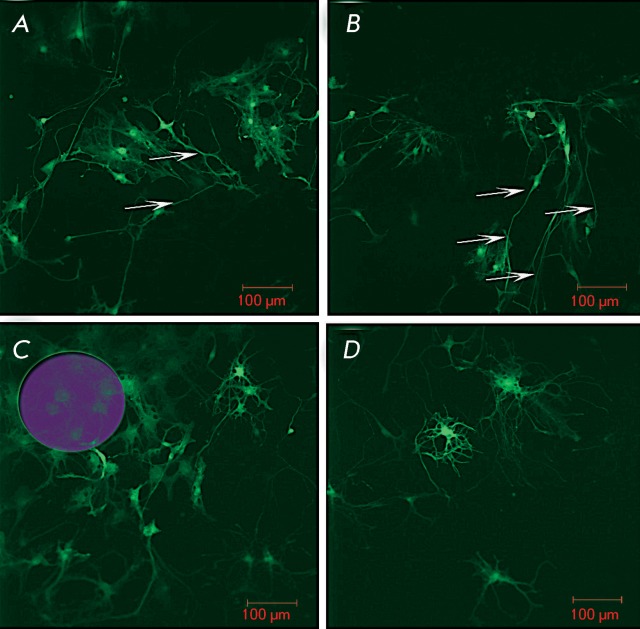
NSPC taxis directed towards the laser-damaged retinal explant area. ( *A, B* ) – Neurite outgrowth (marked with arrows) from transplanted EGFP+ cells following the direction of the vector from the injection site to the laser-damaged area. ( *C, D* ) – Localization of the transplanted NSPCs in the laser-damaged area on day 7 after the transplantation and the development of the dense neurite net on day 14.

The tissue macrostructure within the beam focus changed distinctly after damage with a laser pulse. This was accompanied by the darkening of the cell cytoplasm, an intense vacuolization of the cells, and the coagulation of the intercellular matrix. A delayed effect of laser irradiation (mass cell death in the area of retinal explant damage) was observed on days 2–3 after the injury. A zone of mass cell death with a radius of up to 500 µm formed around the immediate area of laser impact (100 × 100 µm). Thus, the laser-induced damage (irreversible) could clearly be visualized, manifesting itself in an essential reorganization of the intercellular matrix (coagulation), thus resulting in the loss of cellular interactions and in changes to the cell morphology, finally culminating in cell death.

NSPCs and MMSCs from EGFP-positive mice were injected into the centre of explant cell location in order to simulate reparation of the area of the retina damaged by the laser pulse. After the transplanted cells reached their new micro-surrounding, they actively migrated and changed in morphology, sprouting long branching outgrowths, and acquiring neuron-like phenotypes.

**Fig. 3:**
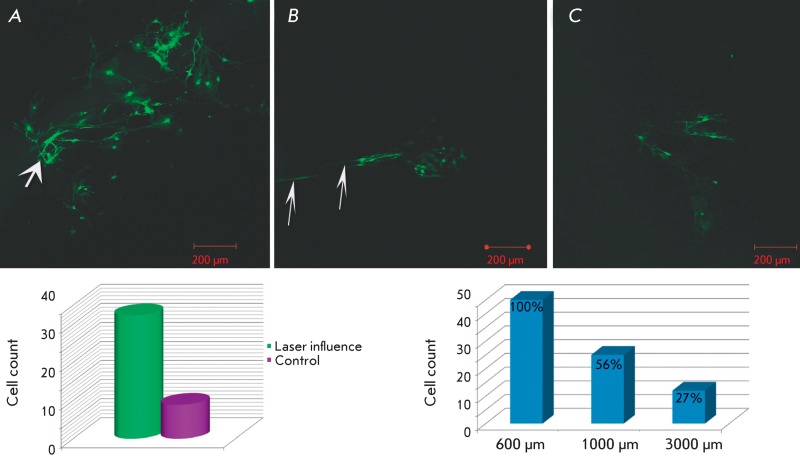
Dynamics of NSPC fast migration to the laser-damaged retina area after the transplantation at a distance of 600 µm ( *A* ), 1000 µm ( *B* ), and 3000 µm ( *C* ) from the laser influence. The diagrams of quantitative differences between cell migration in the damaged and intact explants and the histogram of the percentage of cell count in laser-damaged areas at different distances from the damage site.

The distribution of EGFP-positive cells within the damaged area and the injection area over their migration vector was analyzed during the first 24 h and on day 3 and day 7 after the transplantation. It was established via a comparison of the distribution of the EGFP-positive cells that the most active cell migration occurs from the transplantation site to the damaged area ( *Fig. 2A, B* ). It was shown by daily registration of cell migration by confocal microscopy that the migration was most intense during the first 24 h, followed by a considerable decrease as the cells underwent morphological differentiation on day 3 following the injection. The first cells that migrated to the laser-influenced area were detected 1 h after the transplantation, when they were introduced at a distance of 100 µm. When the cells were transplanted at a distance of 500 and 1000 µm away from the damaged site, they were detected after 12 h or 3–5 days, respectively. Cell migration to the damaged area was most active during the first 3 days after the retinal defect, followed by an abrupt decrease in the level of cell migration. It appeared that 3 days after the transplantation, the injected cells actively migrated to distances of over 1000 µm from the injection site. It is notable that the cells moved towards the damaged area. The injected cells propagated in the explant along a vector directed toward the injured area at a distance of 5 mm, and in the opposite direction, at a distance not exceeding 1 mm ( *Fig. 2C, D* ). A significantly greater ( *p * < 0.01) amount of the transplanted cells was detected in this region via statistical processing of the results of a count of ЕGFP+-NSPCs within one vision field in the damaged area and at the same distance along a vector passing through the injection site ( *Fig. 3* ). 

The propagation of injected cells in the control explants subjected to cell transplantation without laser damaging was uniform in all directions; cell migration from the explant center along the migration routes was predominant. Thus, nonuniformity of the distribution of the transplanted NSPCs with respect to the injected site after the laser-induced damage (the taxis directed towards the defect area) was demonstrated. Similar data concerning the changes in the behavior of injected cells in the neuronal micro-surrounding were obtained after the *in vivo * transplantation of NSPCs [6]. 

After reaching the damaged area, cell migration ceased; formation of asynaptic dendrites propagating in all planes and aggregation of the transplanted cells were observed. The cells that migrated into the damaged area remained there throughout the entire experiment (up to 30 days after the transplantation) and formed a dense neurite network, thereby completely losing their migration activity.

The cell concentration upon transplantation had a considerable impact on the behavior of the transplanted cells. The introduction of single cells into the explant resulted in a rapid end to their migration within the retina and was accompanied by weak morphological differentiation. Single NSPCs acquired a glial phenotype and had glial differentiation markers (GFAP) starting from day 5 after the transplantation. The maximum time during which single MMSCs were retained in the explant was 24 h. The migration activity of cells was suppressed upon transplantation of 50–100 cells. Association was observed, and cell taxis occurred in opposite directions. Only single cells migrated to considerable distances. The situation was different with the transplantation of over 1,000 cells. In this case, most cells actively migrated towards the damaged area, where they formed long neurites, which facilitated the migration of other cells. The differentiation of NSPCs was also faster in comparison to that for the intact explant; the neuronal component was predominant, and the transplanted cells expressed βIII-tubulin and GP-45. The injected cells within the explant not only remained viable for over 2 months, but also actively proliferated.

When studying the distribution of the transplanted NSPCs over the explant with several sequential laser-damaged areas located at different distances from the injection site (600, 1 000, and 3 000 µm), we found that lodging of all damaged areas with the transplanted cells occurred throughout the 7 days immediately following the injection ( *Fig. 3* ). However, the amount of cells in the damaged areas decreased with increasing distance from the injection site. Thus, 56% of the cells detected in the area at a distance of 600 µm were located in the damaged area at a distance of 1 000 µm from the transplantation site, whereas the zone at a distance of 3 000 µm contained only 27% of the cells. 

Two methods of cell introduction were used for the transplantation of MMSCs and NSPCs: firstly, direct injection into the middle area of the explant excrescence margin; secondly, coating of the surface with cells. In the first case, the transplanted cells were in immediate contact with the neuronal component, whereas the *in vivo * suprachoroidal injection was simulated in the second case. After NSPCs were coated onto the surface of the retinal explant, they manifested an almost complete absence of migration activity and neuronal differentiation processes (negative βIII-tubulin staining). This observation allows one to interpret the insignificant therapeutical effect of the transplanted NSPCs in clinical practice after suprachoroidal or retrobulbar injection [7]. After NSPCs were injected deep into neuroretinal layers, the transplanted cells actively migrated and changed their morphology. They sprouted long branching outgrowths, acquired a neuron-like phenotype, and were positively stained against βIII-tubulin and GP-45. 

**Fig. 4:**
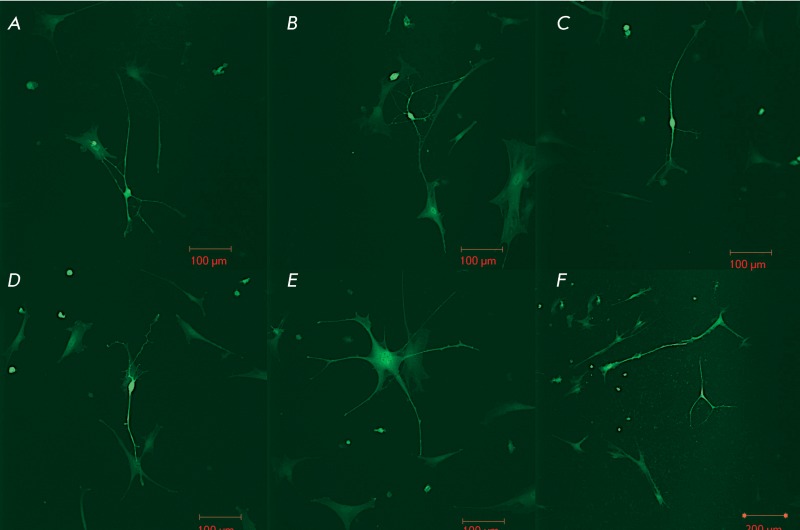
Changes in the morphology of the transplanted MMSCs in laser-damaged retinal explants. The injected MMSCs acquired a neuronal morphology *; (E, F) – * Differentiation of the injected MMSCs according to the glial phenotype.

Rapid migration of individual cells of small diameter (10–15 µm) and the formation of long neurite-like outgrowths and lamellopodia were observed when MMSCs were introduced using both methods. Thus, the transplanted cells were morphologically differentiated according to the new micro-surrounding; the transplanted cells acquired a neural cell phenotype ( *Fig. 4* ). When transplantation of at least 500 cells was performed, MMSCs remained intact within the explant for 30 days. The transplanted MMSCs acquired two characteristic phenotypes: the neuronal one (with long and thin branching outgrowths with ampullary dilatations and a compact cell body) and glial one (with cytoplasmic outgrowths (lamellopodia) and a large nucleus with easily-observable nucleoli). Neurite-like MMSC outgrowths penetrated deep into the retinal explant, formed anastomoses, and were in contact with other transplanted cells and neurons of the explant; i.e., the behavior of MMSCs transplanted into the surrounding of the neuroretina was similar to that of the injected NSPCs. However, in contrast to NSPCs, the method of introduction of MMSCs had no effect on the migration activity of the transplanted cells. MMSCs migrated in all directions from the injection site during the first day, both after being coated onto the surface and after injection into the explant. The onset of cell differentiation resulted in the end of migration, attesting to the fact that the first hours after the transplantation are significant for the migration of the injected cells and the occupation of new niches, as well as their proliferation and differentiation, with the subsequent possibility of defect reparation [8]. 

When the explant was damaged, the changes in MMSC morphology were more rapid in comparison with those in the cells introduced into the control specimens that were not damaged by a laser. In this experimental series, the morphological changes in MMSCs manifested themselves 24 h after the injection, whereas the period for the control specimens was 3 days. These data allow one to assume that the regulatory factors releasing as a result of cell death in the damaged area are of extreme importance for the morphological differentiation of the transplanted cells, which satisfies the conditions of their new micro-surrounding.

**Fig. 5:**
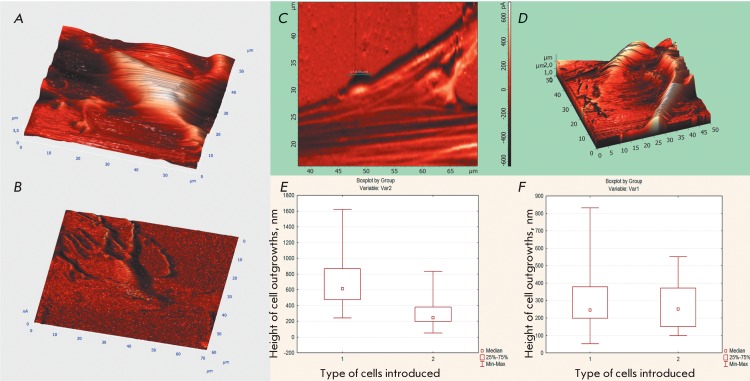
AFM analysis of the surface of the injected MMSCs in the complete contact and semi-contact modes. ( *A* ) – Reconstruction of the MMSC surface after the changes in morphology. ( *B* ) – Early cell migration from the retina explant with lamellopodia formation. ( *C, D* ) – The formation of synaptic connections between the transplanted MMSCs and retinal cells. ( *E* ) – quantitative analysis of the distribution of outgrowths heights: group 1 – glial cells of the retinal explant, group 2 – outgrowth of the transplanted MMSCs ( *p*  < 0.01). ( *F* ) – quantitative analysis of the distribution of outgrowth heights: group 1 – neuronal explant cells, group 2 – outgrowths of the transplanted MMSCs ( *p* > 0.01).

By means of atomic force microscopy, it was possible to show the morphological transformation of MMSCs transplanted into the cells with a neuronal phenotype, their active migration during a period up to 3 days after the injection, and the formation of bipolar and multipolar neurite-like outgrowths. Cell migration from the retinal explant was preceded by lamellopodia formation. AFM was used to measure the heights of lamellopodia, to study their spatial distribution, and to estimate the roughness of their surface. According to the AFM data, the average length of the primary lamellopodia of the migrating cells was 10.1 ± 2.0 µm; the average diameter was equal to 3.6 ± 0.5 µm. An increase in the extent of asynaptic dendrites sprouted by the migrating cells of the retinal explant was observed by day 7 of cell culturing. According to the AFM data, their average length was 21.7 ± 5.0 µm; the average diameter was equal to 0.8 ± 0.23 µm. The neurite-like outgrowths of MMSCs (being several millimeters long and up to 0.47 µm in diameter) propagated on the surface of glial (GFAP-positive) and endothelial (GSL-IB4-immunopositive, GFAP-immunonegative) cells ( *Fig. 5A, B* ). 

The quantitative analysis of the distribution of outgrowth heights for the groups of glia-like cells and neuron-like MMSCs revealed a significant divergence ( *p*  < 0.01) in the heights of the outgrowths formed by glial and endothelial retinal components and the neuronal outgrowths and transplanted MMSCs ( *Fig. 5E* ). It was clear that no statistically significant differences existed ( *p* = 0.52) between the root-mean-square roughness of the surface of the transplanted MMSCs (whose morphology changed to neuron-like) and retinal neurons. No statistically significant differences ( *p * = 0.26) were revealed when comparing the asymmetry of distribution of the outgrowths of the transplanted cells and retinal explant neurons ( *Fig. 5F* ). When examining the images of the cell surface, no statistically significant differences in the range of outgrowth heights were detected between MMSCs and retinal neurons, whereas there were statistically significant differences ( *p * < 0.01) between the heights of MMSC outgrowths and the heights of the outgrowths of glial and endothelial retinal cells. AFM images of synaptic dilatations formed by the transplanted MMSCs on the ends of neurite-like outgrowths were obtained ( *Fig. 5C* ). The interaction between these synapses and explant cells was also studied. The morphometric parameters of the synapses between the transplanted MMSCs and retinal neurons did not differ from the parameters between the transplanted NSPCs and the recipient cells. 

The retinal explant cells were stained with dye Di-I, which was used to demonstrate that no fusion of the transplanted EGFP-positive MMSCs and the retinal cells took place, since no cells carrying both labels were detected.

Based on the data obtained, it can be reasonably suggested that direct introduction of NSPCs into the neuroretina is the optimal method for efficient reparation of retinal damage using cell transplantation. MMSCs, which are capable of initiating and maintaining the reparation processes in the recipient tissue and of following the neuronal differentiation path, can be considered a similar and available alternative to these cells [8]. However, the questions regarding the functional replacement of the missing nerve cells with transplanted MMSCs and their descendants still remain open.

## CONCLUSIONS

The obtained retinal explant cultures are equivalent to * in vivo* neuroretina. 

After MMSCs and NSPCs were injected, active migration of these cells was observed during the first hours after the transplantation. The migration rates were different in the case of injection deep inside the neuroretinal layers and upon application onto the explant surface.

The micro-surrounding of the introduced cells is the key factor for the differentiation of the transplanted cells. In some cases, MMSCs transplanted into the retina may acquire a neuronal phenotype.

A laser can be used to control *in vitro* retinal damage, which stimulates the migration of the transplanted cells towards the injured area and accelerates their differentiation, according to the niche they occupy. 

## Abbreviations

MMSC: bone marrow-derived mesenchymal stem cells
NSPC: neural stem/progenitor cells
EGFP: enhanced green fluorescent protein
